# Atmospheric and Geogenic Controls of Rainwater and Stream-Water Chemistry in an Aleppo-Pine-Dominated Mediterranean Karst Catchment

**DOI:** 10.3390/plants15172611

**Published:** 2026-08-27

**Authors:** Lukrecija Butorac, Ivan Limić, Lucija Lovreškov, Maja Veršić Bratinčević, Goran Jelić, Tamara Jakovljević

**Affiliations:** 1 Institute for Adriatic Crops and Karst Reclamation, Put Duilova 11, 21000 Split, Croatia; ivan.limic@krs.hr (I.L.); maja.versic.bratincevic@krs.hr (M.V.B.); 2 Croatian Institute of Public Health, Borongajska cesta 83 g, 10000 Zagreb, Croatia; lucija.lovreskov@hzjz.hr; 3 Croatian Forest Research Institute, Cvjetno Naselje 41, 10450 Jastrebarsko, Croatia; tamaraj@sumins.hr

**Keywords:** marine aerosols, biogenic inputs, nutrient cycling, seasonal variability, hydrochemical dynamics

## Abstract

This study investigated the hydrochemical linkage between rainwater and stream water in an Aleppo pine-dominated Mediterranean karst catchment in Croatia during 2020–2022, focusing on ion sources, seasonal patterns, and processes governing the transformation of atmospheric inputs into stream-water chemistry. Rainwater reflected mixed atmospheric inputs, with sea-salt fractions accounting for 94.8% of Cl^−^ and 97.9% of Mg^2+^, whereas 97.5% of Ca^2+^ and 79.2% of K^+^ originated from non-sea-salt sources. Stream water was substantially more alkaline and mineralised, with a higher pH (8.23 vs. 6.53), electrical conductivity (327 vs. 19.63 µS cm^−1^) and Ca^2+^ concentrations (65.08 vs. 6.22 mg L^−1^). These differences reflect carbonate weathering during subsurface flow, whereby the dissolution of calcite and dolomite releases Ca^2+^ and Mg^2+^, increases alkalinity and buffering capacity, and results in approximately tenfold-higher stream-water Ca^2+^ and Mg^2+^ concentrations. Seasonal variability was evident, with ion enrichment during low-flow conditions and dilution during wetter periods. Limited synchronous hydrochemical correspondence between rainwater and stream water indicates that atmospheric inputs are modified during catchment transport. Overall, atmospheric deposition provides the initial hydrochemical signal, whereas carbonate weathering, hydrological flow paths, and seasonal biogeochemical processes are the predominant controls on stream-water chemistry.

## 1. Introduction

Mediterranean karst landscapes developed on carbonate bedrock are highly dynamic and environmentally sensitive terrestrial systems [1,2]. In Mediterranean regions, this vulnerability is further enhanced by thin soils, rapid drainage, strong surface groundwater interactions and a highly seasonal hydroclimatic regime, all of which increase the sensitivity of these systems to atmospheric deposition and hydrological change [3,4]. Nearly half of Croatia is underlain by karst and its highly permeable aquifers constitute major sources of drinking water. Consequently, understanding the hydrochemical processes governing these environments is of particular importance for sustainable water-resource management [5]. Together, these geological, climatic, and ecological characteristics create a hydrologically dynamic environment in which atmospheric deposition, rapid infiltration, and limited surface runoff strongly regulate water–rock interactions and determine the chemical composition of rainwater and stream water [6].

In such environments, atmospheric deposition represents an important pathway through which natural and anthropogenic substances are transferred from the atmosphere to terrestrial and aquatic ecosystems [7]. In forested catchments, this transfer is substantially modified by canopy processes, including interception, retention of dry deposition, canopy leaching and throughfall, which alter both the quantity and chemical composition of water reaching the forest floor [8,9]. These processes are especially relevant in Mediterranean coastal regions, where atmospheric inputs typically reflect a mixture of marine aerosols, continental dust, biogenic material and anthropogenic emissions [10]. Consequently, rainwater chemistry integrates the influence of multiple atmospheric sources and transformation processes occurring during atmospheric transport, providing valuable information on the origin and evolution of dissolved ions [11].

Major ions in rainwater originate from multiple, often overlapping sources. Marine aerosols are the principal source of Na^+^, Cl^−^, Mg^2+^ and part of SO_4_^2−^, whereas continental dust provides an important source of non-sea-salt Ca^2+^ and Mg^2+^ [12,13]. Anthropogenic sources such as agricultural NH_3_ emissions and NO_x_ released by traffic, combustion and industry contribute to NH_4_^+^ and NO_3_^−^ concentrations. Non-sea-salt SO_4_^2−^ and, to a lesser extent, K^+^ may also originate from secondary atmospheric production and biomass burning [14]. Biogenic inputs may also contribute to potassium concentrations in rainfall. In forest ecosystems, spring pollen release can substantially increase atmospheric K^+^ deposition owing to the high biomass of pollen and its potential for long-distance transport, thereby complicating source apportionment, particularly in Mediterranean environments where biological activity exhibits pronounced seasonal variability [15,16]. In Mediterranean environments, the relative importance of these sources varies seasonally owing to changes in air mass transport, rainfall regime, drought duration, and emission intensity, producing distinct seasonal patterns in rainwater chemistry [14,17].

In karst catchments, however, atmospheric inputs contained in rainwater are rarely transferred directly to stream water. Instead, stream-water chemistry reflects the combined influence of atmospheric deposition, carbonate weathering, hydrological flow paths, residence time, and biogeochemical processing within the catchment [4,18]. During subsurface flow, carbonic acid, formed by the dissolution of atmospheric and soil CO_2_ in water, promotes the dissolution of calcite (CaCO_3_) and dolomite (CaMg(CO_3_)_2_), releasing Ca^2+^, Mg^2+^ and bicarbonate (HCO_3_^−^) ions into the solution. These reactions progressively increase alkalinity, pH and electrical conductivity, thereby largely determining the hydrochemical characteristics of Mediterranean karst streams [19,20]. Consequently, stream-water chemistry in carbonate catchments is typically characterised by elevated Ca^2+^ and Mg^2+^ concentrations, high alkalinity, and enhanced buffering capacity relative to precipitation. These interactions are particularly evident in Mediterranean environments, where wetter winter periods alternate with prolonged summer drought. During dry periods, atmospheric solutes accumulate on canopy and soil surfaces and are subsequently mobilized by rainfall, generating flushing pulses to soils and streams [21]. Conversely, high stream discharge promotes dilution and rapid hydrological transfer, whereas prolonged low-flow conditions enhance mineral dissolution through longer water residence times and stronger water–rock interactions [4,22].

These hydrochemical processes are especially relevant in Mediterranean forested catchments exposed to elevated atmospheric nitrogen and sulphur inputs. Although base cations contribute to acid neutralization and support ecosystem functioning, reactive nitrogen and sulphur deposition may alter nutrient cycling, promote acidification and affect the hydrochemical functioning of sensitive catchments [23,24]. Studies on Mediterranean forests along the eastern Adriatic coast indicate that inorganic nitrogen deposition can approach or exceed critical loads in some forest types, thereby highlighting the vulnerability of these ecosystems to eutrophication and related chemical change [9,25]. Therefore, understanding how atmospherically derived solutes are transformed during their transport from rainfall to stream water remains one of the key challenges in Mediterranean karst hydrogeochemistry.

Despite the recognized importance of atmospheric deposition in Mediterranean environments, the transfer of atmospherically derived solutes from rainwater to stream water in karst catchments remains insufficiently understood. This knowledge gap is particularly evident in small Mediterranean karst catchments, where hydrochemical responses are strongly influenced by carbonate weathering, seasonal hydrology and in-catchment transformation processes. Nevertheless, relatively few studies have simultaneously examined rainwater and stream-water chemistry within the same catchment over extended monitoring periods. Consequently, the extent to which rainfall derived solutes are transformed by hydrological, geochemical, and biogeochemical processes before being expressed in stream-water chemistry remains poorly constrained. Such systems are particularly suitable for detecting environmental changes because they respond rapidly to variations in atmospheric inputs, although interpreting these responses requires continuous chemical monitoring across contrasting seasonal conditions [26]. The Žrnovnica catchment, a Mediterranean karst basin dominated by Aleppo pine (*Pinus halepensis* Mill.) forest, provides an appropriate natural laboratory for investigating these interactions.

To address this knowledge gap, unlike previous Mediterranean hydrochemical studies, which have generally focused on either atmospheric deposition [4,9,10,17] or surface-water chemistry [27,28,29], the present study simultaneously examined rainwater and stream-water chemistry within the same Mediterranean karst catchment over a three-year monitoring period. This integrated monitoring approach directly links atmospheric deposition to the hydrochemical signature of stream water, allowing atmospherically derived solutes to be distinguished from those modified by carbonate weathering, hydrological flow paths, and biogeochemical processes. Consequently, this approach provides new insights into the processes governing the transformation of atmospheric inputs before they are reflected in stream-water chemistry.

Based on the current understanding of hydrochemical processes in Mediterranean karst catchments, we hypothesized that although atmospheric deposition represents an important external source of dissolved ions, its hydrochemical signature is substantially modified during transport through the catchment by carbonate weathering, hydrological flow paths, and biogeochemical processes before reaching stream water. Consequently, stream-water chemistry was hypothesized to reflect stronger geogenic control than direct atmospheric inputs, while seasonal hydrological conditions were expected to regulate the magnitude of this transformation.

To test this hypothesis, the aims of this study were to: (1) characterize the chemical composition of rainwater and stream water, (2) identify the dominant sources of major ions, (3) evaluate the extent to which atmospheric deposition is reflected in stream-water chemistry, and (4) assess seasonal variability in both water types during a three-year monitoring period (2020–2022).

## 2. Results

### 2.1. Chemical Composition of Rainwater and Stream Water

Rainwater and stream water differed markedly in their physicochemical properties and ionic composition throughout the 2020–2022 study period (Table 1). Rainwater was slightly acidic to neutral (pH 5.22–7.92; median 6.53), weakly mineralized (median EC 19.63 µS cm^−1^) and low total alkalinity (median 0.34 mg L^−1^). In contrast, stream water was consistently alkaline (pH 7.46–8.99; median 8.23), substantially more mineralized (median EC 327 µS cm^−1^), and showed markedly higher total alkalinity (median 3.40 mg L^−1^) (Table 1).

Among the major ions, Ca^2+^ and Cl^−^ predominated in rainwater, whereas stream water was characterised by substantially higher concentrations of Ca^2+^, Cl^−^, Na^+^, SO_4_^2−^, and Mg^2+^. The largest differences between the two water types were observed for Ca^2+^ and Mg^2+^, whose median concentrations were approximately ten times higher in stream water than in rainwater. In contrast, NH_4_^+^ and NO_3_^−^ occurred at comparatively low concentrations in both water types and did not differ significantly, whereas all remaining physicochemical parameters and major ions showed statistically significant differences (*p* < 0.05) (Table 1).

**Figure 1:**
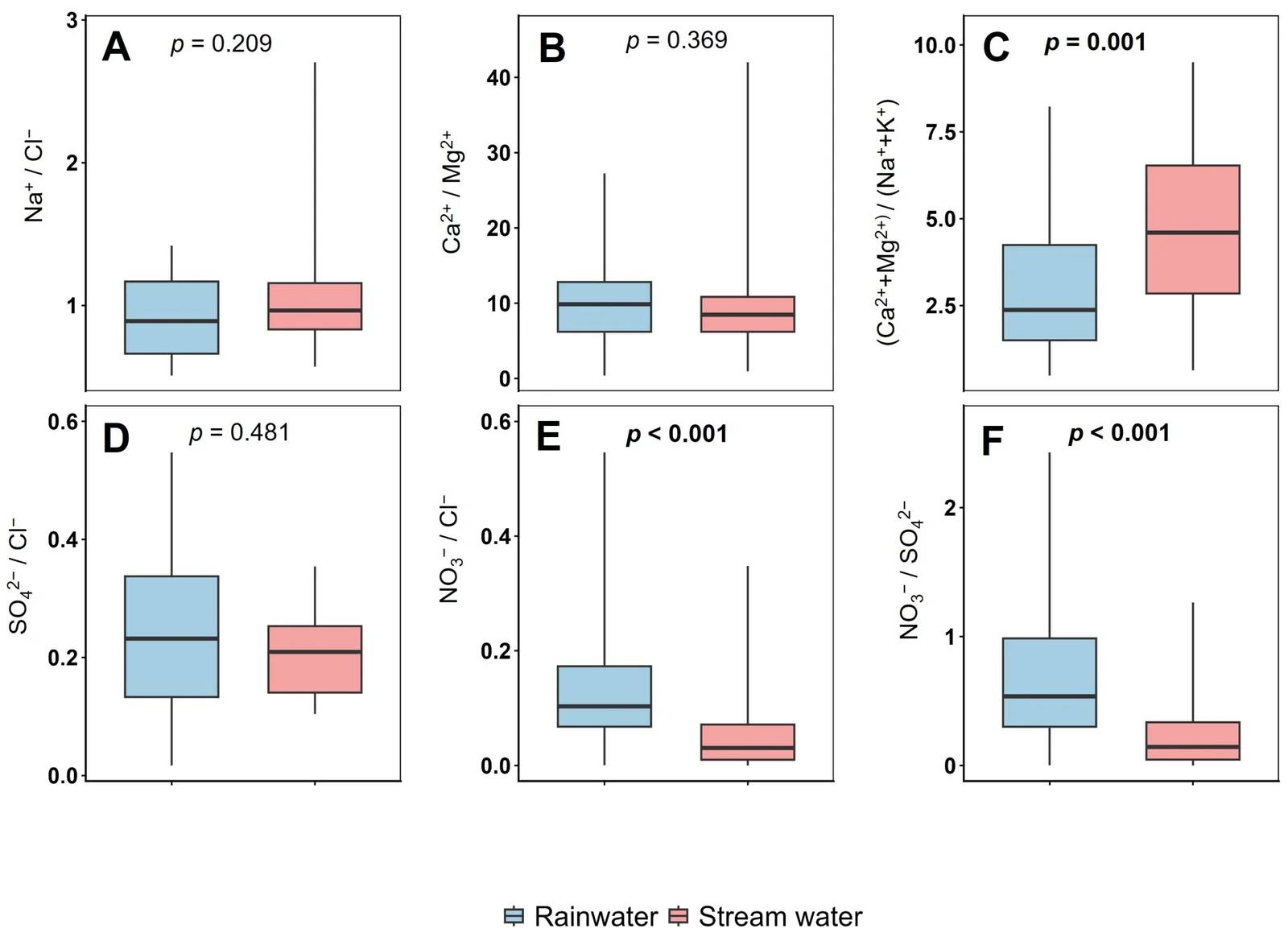
Comparison of ion ratios between rainwater and stream water during the study period (2020–2022). Boxes represent the interquartile range (Q1–Q3): (**A**) Na^+^/Cl^−^; (**B**) Ca^2+^/Mg^2+^; (**C**) (Ca^2+^ + Mg^2+^)/(Na^+^ + K^+^); (**D**) SO_4_^2−^/Cl^−^; (**E**) NO_3_^−^/Cl^−^, and (**F**) NO_3_^−^/SO_4_^2−^ ratios. Horizontal lines within the boxes indicate the median, and whiskers extend to the minimum and maximum values. Statistical differences between rainwater and stream water (Mann–Whitney U test, *p* < 0.05) are shown in bold. Ratios are expressed in molar units.

Ion ratios further highlighted the contrasting hydrochemical characteristics of rainwater and stream water (Figure 1). In rainwater, the Na^+^/Cl^−^ ratio (median 0.890) closely matched the seawater reference value (0.859), confirming the dominant influence of marine aerosols. In contrast, stream water showed a significantly higher (Ca^2+^ + Mg^2+^)/(Na^+^ + K^+^) ratio (median 4.598; *p* < 0.001), whereas the NO_3_^−^/Cl^−^ and NO_3_^−^/SO_4_^2−^ ratios were significantly lower than in rainwater, highlighting contrasting hydrochemical characteristics of the two water types (Figure 1).

**Figure 2:**
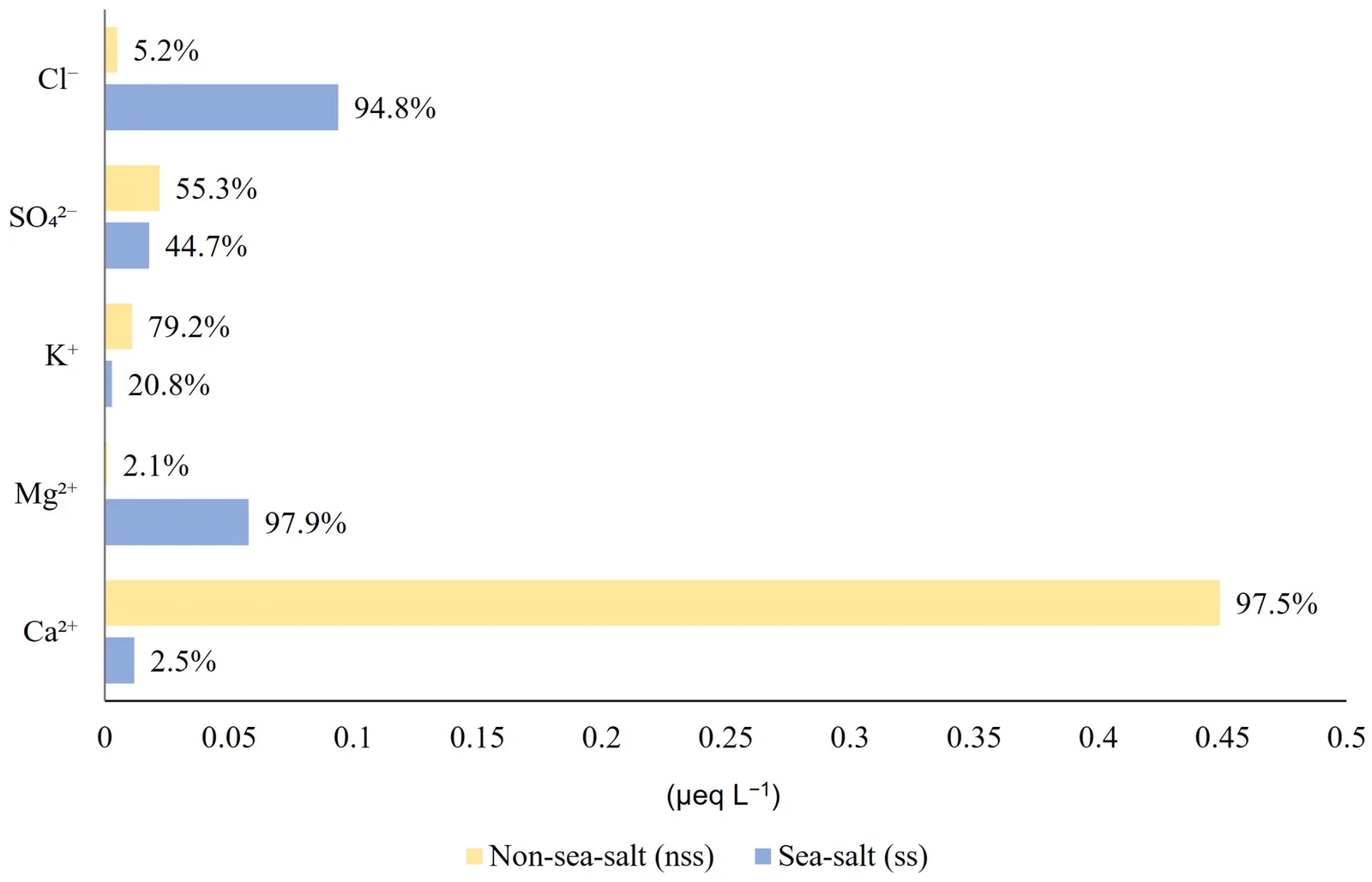
Sea-salt (ss) and non-sea-salt (nss) contributions to the ionic composition of rainwater based on seawater reference ratios. Bars represent the concentrations (µeq L^−1^), whereas the percentages shown beside the bars indicate the relative contribution of each fraction to the total concentration of individual ions.

Sea-salt (ss) and non-sea-salt (nss) fractionation further distinguished marine and continental contributions to rainwater chemistry (Figure 2). Chloride and Mg^2+^ were predominantly of marine origin, with sea-salt contributions of 94.8% and 97.9%, respectively, whereas Ca^2+^ and K^+^ were largely derived from non-sea-salt sources (97.5% and 79.2%, respectively). Sulphates showed a mixed origin, with a slight predominance of the non-sea-salt fraction (55.3%).

### 2.2. Seasonal Variability in Rainwater and Stream-Water Chemistry

**Figure 3:**
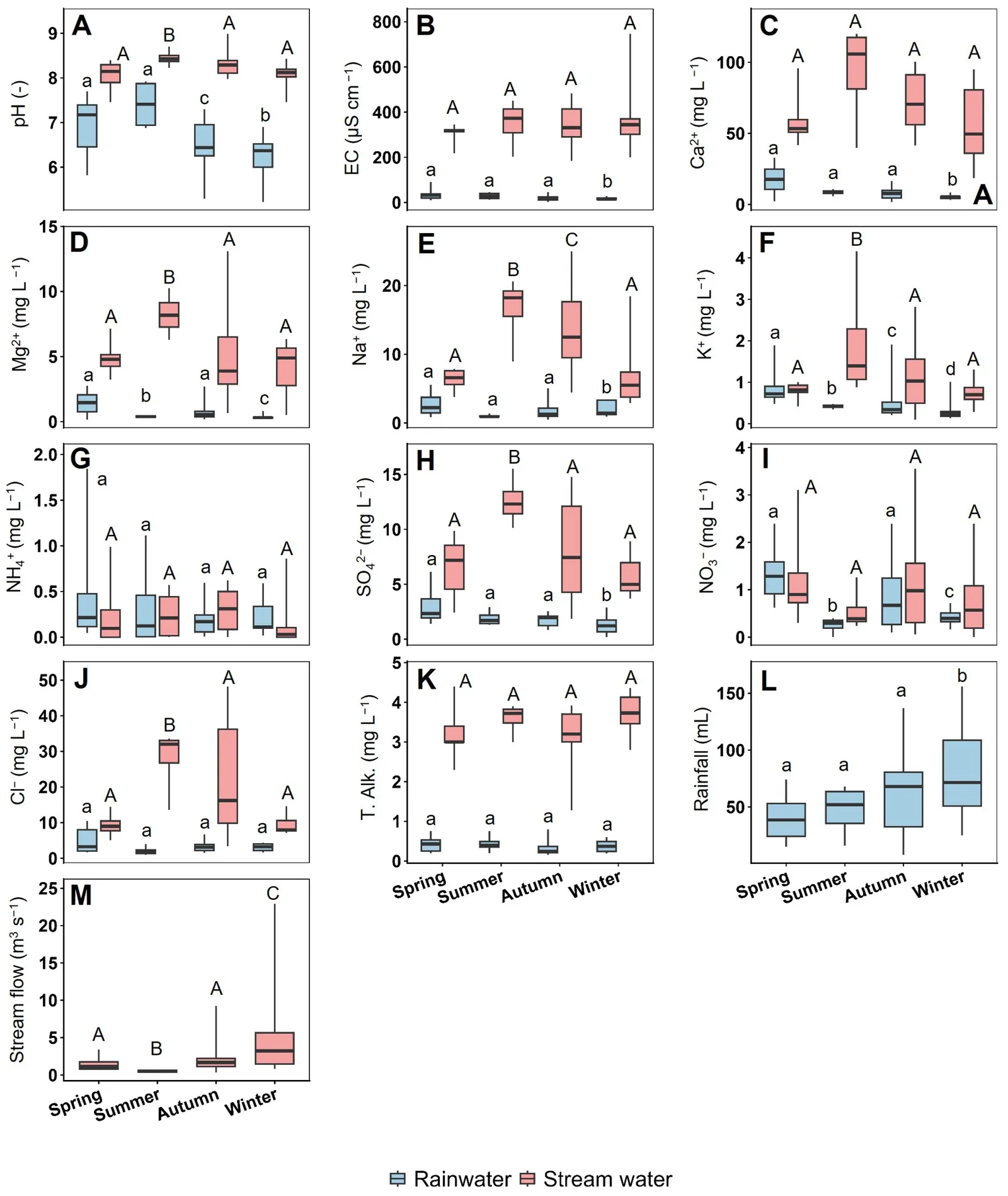
Seasonal variability in physicochemical parameters, major ion concentrations, rainfall amount, and stream flow during the study period (2020–2022). Boxes represent the interquartile range (Q1–Q3): (**A**) pH; (**B**) electrical conductivity (EC); (**C**) Ca^2+^; (**D**) Mg^2^; (**E**) Na^+^; (**F**) K^+^; (**G**) NH_4_^+^; (**H**) SO_4_^2−^; (**I**) NO_3_^−^; (**J**) Cl^−^; (**K**) total alkalinity (T. Alk.); (**L**) rainfall amount, and (**M**) stream flow. Horizontal lines within the boxes indicate the median, and whiskers extend to the minimum and maximum values. Lowercase letters (a–d) indicate statistically significant differences among seasons for rainwater, whereas uppercase letters (A–C) indicate statistically significant differences among seasons for stream water (Kruskal–Wallis test, *p* < 0.05).

Most measured parameters showed clear seasonal variation (Figure 3). Rainfall amounts were highest during winter, whereas rainwater was generally more mineralised during spring and summer, as indicated by higher EC values and elevated concentrations of most major cations. Among the major ions, Ca^2+^, Mg^2+^, Na^+^, and K^+^ reached their highest concentrations in spring, while SO_4_^2−^ and NO_3_^−^ showed their lowest concentrations during winter. In contrast, NH_4_^+^ concentrations and total alkalinity did not vary significantly among seasons (Figure 3).

**Figure 4:**
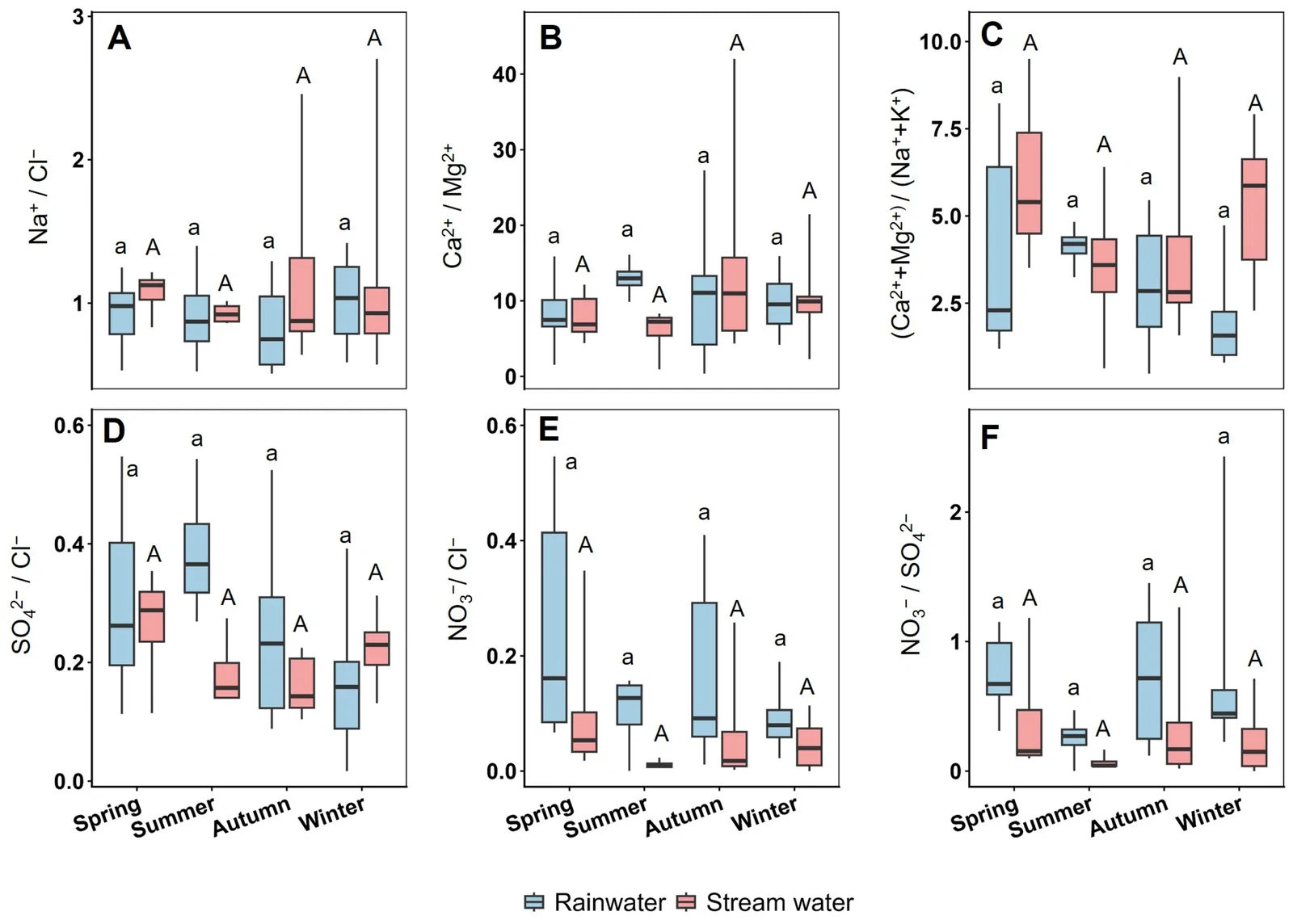
Seasonal comparison of ion ratios in rainwater and stream water during the study period (2020–2022). Boxes represent the interquartile range (Q1–Q3): (**A**) Na^+^/Cl^−^; (**B**) Ca^2+^/Mg^2+^; (**C**) (Ca^2+^ + Mg^2+^)/(Na^+^ + K^+^); (**D**) SO_4_^2−^/Cl^−^; (**E**) NO_3_^−^/Cl^−^, and (**F**) NO_3_^−^/SO_4_^2−^ ratios. Horizontal lines indicate the median, and whiskers extend to the minimum and maximum values. Lowercase letters indicate statistically significant differences among seasons for rainwater, whereas uppercase letters indicate statistically significant differences among seasons for stream water (Kruskal–Wallis test, *p* < 0.05).

Seasonal patterns in stream water differed from those observed in rainwater. Stream flow reached its maximum during winter and minimum during summer, whereas stream-water pH was slightly higher during summer. Concentrations of Na^+^, K^+^, Mg^2+^, Cl^−^ and SO_4_^2−^ also peaked during summer, while Ca^2+^, NO_3_^−^, NH_4_^+^ and EC showed no significant seasonal variation (Figure 3). Seasonal differences in ion ratios were also evident (Figure 4). In rainwater, SO_4_^2−^/Cl^−^ ratios were highest during summer, whereas NO_3_^−^/Cl^−^ ratios peaked in spring. A similar seasonal pattern was observed in stream water, where both SO_4_^2−^/Cl^−^ and NO_3_^−^/Cl^−^ ratios reached their highest values during spring. Overall, seasonal variability was more pronounced in rainwater than in stream water, although both water types exhibited distinct hydrochemical responses to seasonal hydrological conditions.

### 2.3. Correlations and Multivariate Patterns in Rainwater and Stream Water

Spearman’s rank correlation analysis revealed generally weak relationships between corresponding parameters in rainwater and stream water (Table 2). Over the entire study period, a statistically significant positive correlation was observed only for NO_3_^−^ (ρ = 0.370; *p* = 0.034). Seasonal analysis identified a significant negative correlation for Ca^2+^ during spring (ρ = −0.738, *p* = 0.037) and a significant positive correlation during autumn (ρ = 0.682, *p* = 0.021), whereas no significant seasonal correlations were found for the remaining parameters (*p* > 0.05). Although relatively high correlation coefficients were obtained for Mg^2+^, Na^+^, and SO_4_^2−^ during summer, these results should be interpreted with caution because they were based on only four paired observations.

**Figure 5:**
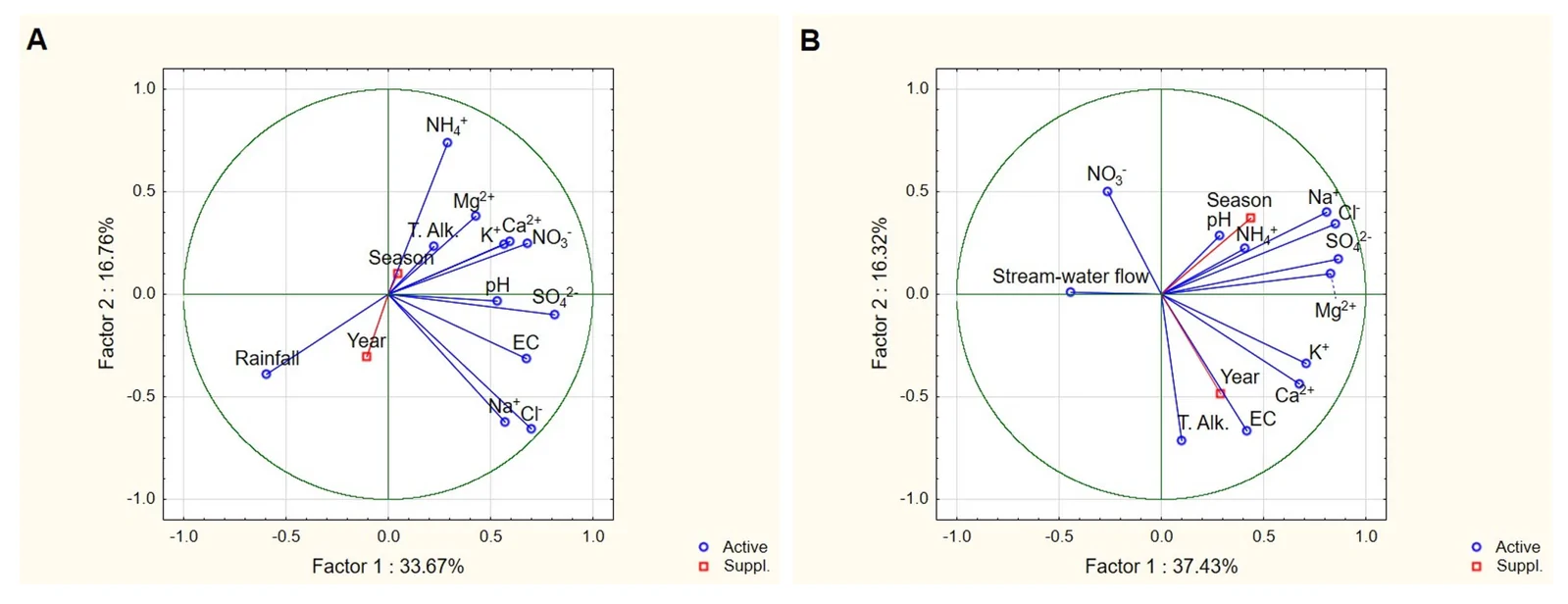
Principal component analysis (PCA) biplots of major ions and physicochemical parameters for (**A**) rainwater and (**B**) stream water during 2020–2022. Vectors represent variable loadings on the first two principal components.

Principal component analysis (PCA) identified the dominant hydrochemical gradients in both rainwater and stream water (Figure 5A,B). In rainwater, four principal components explained 74.2% of total variance. PC1 (33.7%) represented the primary mineralization gradient, characterised by strong positive loadings of SO_4_^2−^, Cl^−^, NO_3_^−^, Na^+^, K^+^, Ca^2+^, Mg^2+^, pH and EC, whereas PC2 (16.8%) distinguished NH_4_^+^–Mg^2+^ associations from Na–Cl–EC relationships. In stream water, four principal components explained 73.8% of the total variance. PC1 (37.4%) represented the dominant mineralization gradient, characterized by high positive loadings of SO_4_^2−^, Cl^−^, Na^+^, Mg^2+^, K^+^, Ca^2+^ and EC, together with a negative association with streamflow. PC2 (16.3%) separated NO_3_^−^, Na^+^, Cl^−^, and pH from alkalinity, EC, Ca^2+^, and K^+^. Seasonal clustering of samples within the PCA ordination indicated distinct seasonal hydrochemical patterns in both rainwater and stream water (Figure 5A,B).

Overall, both the correlation analysis (Table 2) and PCA (Figure 5A,B) consistently demonstrated limited correspondence between rainwater and stream-water chemistry while revealing distinct hydrochemical patterns in the two water types.

## 3. Discussion

### 3.1. Transformation of Atmospheric Inputs from Rainwater to Stream-Water Chemistry

The present study demonstrates that rainwater and stream water represent successive stages of hydrochemical evolution within the Žrnovnica Mediterranean karst catchment rather than two independent water compartments. While rainwater primarily reflects the chemical composition of atmospheric deposition reaching the catchment, stream water integrates the cumulative effects of canopy interactions, groundwater recharge, subsurface transport, water–rock interactions and biogeochemical processes operating within the karst system. Consequently, the pronounced differences in pH, electrical conductivity, alkalinity and major-ion composition between rainwater and stream water (Table 1), together with the weak correlations observed for most variables (Table 2) and the distinct separation of both water types in the PCA (Figure 5A,B), demonstrate that atmospheric deposition alone cannot explain stream-water chemistry. Instead, precipitation provides the initial hydrochemical signal, which is progressively modified during transport through the catchment before being expressed in stream water. Similar hydrochemical evolution has been reported for Mediterranean forested catchments along the eastern Adriatic coast [4,9,25], other Mediterranean karst environments [30,31,32,33], and Croatian carbonate systems [34,35], while the fundamental role of water–rock interactions in controlling natural water chemistry has long been recognised in classical hydrogeochemical studies [18,36,37].

The chemical composition of rainwater indicates that atmospheric deposition in the Žrnovnica catchment originated from a combination of marine, continental, biological and anthropogenic sources (Table 1, Figure 1, Figure 2 and Figure 5A,B). Although the catchment is predominantly characterised by karst terrain and Mediterranean vegetation, previous assessments of the broader Jadro–Žrnovnica catchment identified settlements and domestic wastewater, road traffic, production and business zones, quarrying and other industrial activities, as well as agriculture and livestock farming as potential anthropogenic pressures on groundwater quality [38]. Agricultural activities, including the use of mineral and organic fertilisers and plant-protection products, may represent additional sources of diffuse inputs to the highly permeable karst system [38]. These documented human activities provide an appropriate regional context for interpreting potential anthropogenic contributions to rainwater and stream-water chemistry, although their individual contributions were not quantified in the present study. Marine aerosols represented the dominant atmospheric input, as demonstrated by the Na^+^/Cl^−^ ratio and by the predominance of sea-salt Cl^−^ and Mg^2+^ (Figure 1 and Figure 5A,B). This finding agrees with previous observations from the eastern Adriatic coast [4,9] and with numerous studies from Mediterranean coastal regions [12,13,39,40,41], where sea spray has been identified as a major source of sodium, chloride and magnesium in coastal precipitation. In contrast, the predominance of non-sea-salt Ca^2+^ and K^+^ reflects substantial continental and biological contributions. The high non-sea-salt fraction of calcium is consistent with inputs of carbonate-rich mineral dust derived from local lithology together with episodic Saharan dust intrusions, which have been recognised as important contributors to atmospheric calcium throughout the Mediterranean Basin [14,42,43,44,45]. Higher spring potassium concentrations (Figure 3 and Figure 4) coincide with the flowering period of Aleppo pine, suggesting that enhanced atmospheric pollen deposition may represent additional seasonal source of potassium [15,16]. Sulphate exhibited a mixed marine and non-marine origin, reflecting both sea-salt aerosols and secondary atmospheric sulphur formed from regional anthropogenic emissions, a pattern widely reported for Mediterranean coastal environments [17,46]. Collectively, these observations indicate that rainwater chemistry records the integrated composition of atmospheric inputs reaching the catchment and therefore represents the initial hydrochemical signal entering the terrestrial ecosystem.

Despite this pronounced atmospheric imprint, only a limited proportion of the original hydrochemical signal was conservatively transferred to stream water. Spearman’s correlation analysis revealed a significant positive relationship only for NO_3_^−^ over the entire monitoring period, while Ca^2+^ showed significant but seasonally contrasting relationships, with a negative correlation during spring and a positive correlation during autumn (Table 2). No significant relationships were observed for the remaining physicochemical parameters or major ions. These results indicate that the majority of precipitation-derived solutes undergo substantial transformation during their passage through the catchment rather than being directly transferred to the stream. Such behaviour is characteristic of Mediterranean forested karst catchments, where canopy processes, infiltration through shallow carbonate soils, groundwater flow and biological activity progressively modify the chemical composition of atmospheric deposition before groundwater emerges as streamflow [8,9,18,25,47,48,49]. Consequently, stream-water chemistry should be interpreted as the cumulative outcome of internal catchment processes rather than as a direct reflection of precipitation chemistry.

Nitrogen species provide a particularly informative example of the transformation of atmospheric inputs within the catchment. Although NO_3_^−^ was the only parameter showing a significant overall correlation between rainwater and stream water (Table 2), nitrate concentrations remained consistently low in stream water throughout the monitoring period (Table 1). This behaviour is consistent with substantial retention and transformation of reactive nitrogen within the catchment before it reaches the stream, despite continuous atmospheric deposition. Efficient nitrogen retention has been widely documented in Mediterranean forest ecosystems [9,25,49], whereas the importance of microbial processing and nitrate retention during subsurface transport has been emphasised in previous hydrochemical studies [47,48,50,51,52,53]. Consequently, stream-water nitrate concentrations reflect internal ecosystem nitrogen cycling rather than direct atmospheric inputs, illustrating the capacity of Mediterranean forested catchments to buffer short-term fluctuations in atmospheric nitrogen deposition.

In contrast, calcium and magnesium clearly demonstrate the dominant influence of geogenic processes on stream-water chemistry. Seasonal correlations for Ca^2+^ differed in direction, with a significant negative relationship between rainwater and stream water during spring (ρ = −0.738, *p* = 0.037) and a significant positive relationship during autumn (ρ = 0.682, *p* = 0.021) (Table 2). This contrasting seasonal behaviour further indicates that the relationship between atmospheric Ca^2+^ inputs and stream-water Ca^2+^ concentrations is not conservative, but is strongly modified by seasonally varying hydrological and geochemical processes within the catchment. Moreover, calcium concentrations in stream water remained approximately one order of magnitude higher than those measured in rainwater (Table 1). The markedly higher alkalinity, electrical conductivity (Table 1), together with the higher (Ca^2+^ + Mg^2+^)/(Na^+^ + K^+^) ratios (Figure 1) indicate that carbonate weathering overwhelmingly exceeds direct atmospheric inputs as the principal source of dissolved base cations. Similar hydrochemical evolution controlled by carbonate dissolution has been reported for Mediterranean karst catchments [30,31,32,33,34,35,54], while the underlying hydrogeochemical mechanisms are consistent with the classical conceptual models of natural water chemistry [18,36,37]. Rather than preserving the chemical composition of precipitation, infiltrating water progressively acquires dissolved constituents during groundwater flow through carbonate bedrock, producing the characteristic hydrochemical signature observed in stream water.

The principal component analysis provides an integrated interpretation of these hydrochemical processes by linking physicochemical parameters, major ions and hydrological variables within a common multivariate framework (Figure 5A,B). Unlike the correlation analysis, which evaluates relationships between individual variables, PCA identifies the dominant processes controlling overall water chemistry. In rainwater, the first principal component represents an atmospheric mineralisation gradient characterised by marine-derived ions together with non-marine Ca^2+^, K^+^ and SO_4_^2−^, reflecting the combined influence of marine aerosols, mineral dust, biological inputs and secondary atmospheric products. In contrast, stream-water samples are primarily structured along a geogenic gradient dominated by Ca^2+^, Mg^2+^, alkalinity and electrical conductivity, while showing an inverse relationship with streamflow. Similar multivariate separation between atmospheric and geogenic controls has been reported for Mediterranean hydrochemical systems [54,55]. The negative association with streamflow is consistent with the interpretation that groundwater residence time represents an important control on the intensity of water–rock interactions. During low-flow conditions, prolonged subsurface residence is likely to enhance mineral dissolution and stream-water mineralisation, whereas increased recharge during wetter periods probably promotes dilution and reduces the extent of geochemical evolution [31,32,33,56,57,58]. Beyond its hydrochemical significance, this pattern also reflects the ecological functioning of the catchment, where seasonal hydrological connectivity regulates nutrient retention, solute transformation and the buffering capacity of the forested karst ecosystem. The separation of rainwater and stream-water samples in the PCA therefore illustrates not only differences in hydrochemical composition but also the capacity of the ecosystem to modify atmospheric inputs before they are exported from the catchment.

**Figure 6:**
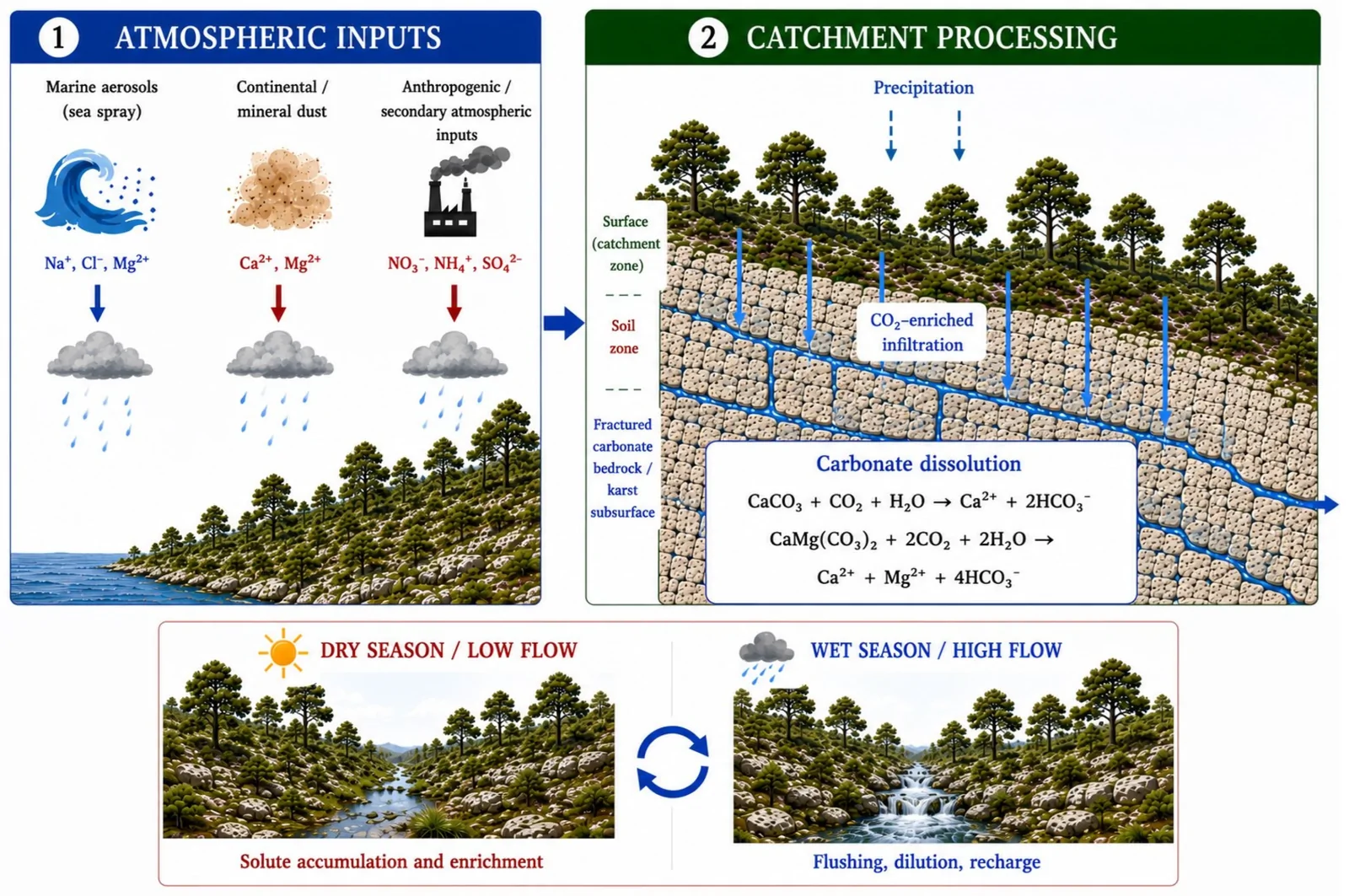
Conceptual model of atmospheric and geogenic controls on rainwater and stream-water chemistry in an Aleppo-pine-dominated Mediterranean karst catchment.

The remarkable agreement among several independent analytical approaches substantially strengthens the proposed interpretation. Major-ion chemistry, ionic ratios, sea-salt/non-sea-salt fractionation, seasonal analyses, correlation analysis and principal component analysis consistently demonstrate that atmospheric deposition supplies the initial hydrochemical signal entering the catchment, whereas canopy interactions, groundwater residence time, water–rock interactions and biogeochemical processes progressively modify this signal before it is expressed in stream-water chemistry. Rather than acting as passive recipients of atmospheric deposition, Mediterranean forested karst catchments function as dynamic hydrochemical filters that regulate both the magnitude and the chemical composition of solute export. These complementary lines of evidence are synthesised in the conceptual model proposed in this study (Figure 6), which illustrates the sequential transformation of atmospheric inputs from precipitation to stream water. The efficiency of these transformation processes, however, is not constant throughout the year but varies according to seasonal hydrological conditions.

### 3.2. Seasonal Hydrological Regulation of Hydrochemical Transformation

Seasonal hydrological variability represents one of the principal controls governing hydrochemical transformation within the Žrnovnica catchment. The Mediterranean climate is characterised by alternating wet winters and prolonged summer droughts, producing pronounced seasonal differences in precipitation, groundwater recharge, stream discharge and subsurface residence time. These hydrological changes regulate both the composition of atmospheric inputs reaching the catchment and the extent to which those inputs are modified during subsurface transport. Consequently, the seasonal patterns observed in both rainwater and stream water (Figure 3, Figure 4 and Figure 5A,B) reflect the combined influence of atmospheric deposition, hydrological connectivity, carbonate weathering and ecosystem processes. Similar hydrological regulation of stream-water chemistry has been recognised in Mediterranean carbonate catchments, where seasonal recharge and groundwater residence time control the intensity of hydrochemical evolution [22,30,31,32,33], while recent studies have further highlighted the importance of hydrological connectivity and flow pathways in regulating seasonal solute transport [56,57,58,59].

Rainwater exhibited substantially greater seasonal variability than stream water (Figure 3 and Figure 4), indicating that precipitation chemistry responded primarily to changing atmospheric conditions. Elevated concentrations of major cations during spring and summer are consistent with the seasonal accumulation of marine aerosols, mineral dust and dry atmospheric deposition during prolonged rain-free periods, followed by their mobilisation during subsequent rainfall events [10,14,17]. The enhanced contribution of mineral dust and Saharan outbreaks previously reported for Mediterranean precipitation [36,37,38,39] provides a plausible explanation for the higher non-marine calcium concentrations observed during these periods. Likewise, the higher spring potassium concentrations are consistent with seasonal pollen release from Mediterranean vegetation [15,16], whereas lower winter concentrations reflect dilution associated with greater rainfall amounts and more efficient atmospheric scavenging [14,17,46]. These observations indicate that seasonal variability in rainwater chemistry is governed primarily by atmospheric processes rather than by catchment-scale controls.

In contrast, stream-water chemistry responded predominantly to seasonal hydrological functioning within the catchment. During summer, reduced stream discharge and prolonged groundwater residence times promoted increased mineralisation, reflected by higher concentrations of Na^+^, Mg^2+^, Cl^−^ and SO_4_^2−^ (Figure 3 and Figure 4). These conditions enhance contact between infiltrating water and carbonate bedrock, thereby increasing the intensity of water–rock interactions and the release of dissolved constituents into groundwater. Comparable seasonal enrichment during low-flow conditions has been reported for Mediterranean carbonate aquifers and streams [22,30,31,32,33], while the influence of residence time on geochemical evolution has been emphasised in recent hydrological studies [56,57,59]. The inverse relationship between streamflow and the dominant hydrochemical gradient identified by the PCA (Figure 5B) further confirms that stream-water chemistry is primarily regulated by internal hydrological processes rather than by short-term changes in precipitation chemistry.

The hydrochemical response during winter was fundamentally different. Increased precipitation and groundwater recharge shortened groundwater residence time, enhanced hydrological connectivity and promoted dilution of dissolved ions, thereby reducing the relative influence of carbonate weathering (Figure 3 and Figure 4). At the same time, rainfall mobilised atmospheric particles accumulated on vegetation and soil surfaces during preceding dry periods, producing short-term flushing of dissolved constituents before dilution became the dominant process. Similar interactions between groundwater renewal, dilution and episodic flushing have been described for Mediterranean karst catchments [22,31,32,33], while process-based studies have demonstrated that seasonal changes in flow pathways strongly regulate both solute transport and hydrochemical evolution [56,57,58]. These contrasting seasonal mechanisms explain why rainwater and stream water exhibited only limited seasonal correspondence despite being exposed to the same climatic forcing.

Overall, the results demonstrate that seasonal hydrology regulates hydrochemical transformation through two complementary mechanisms. First, it controls the seasonal accumulation and mobilisation of atmospheric inputs reaching the catchment. Second, it determines the extent to which these inputs are modified during groundwater transport by regulating residence time, hydrological connectivity and water–rock interactions. Consequently, stream-water chemistry reflects the integrated response of the catchment to seasonal hydrological processes rather than the direct chemical composition of precipitation. This interpretation is supported by the seasonal patterns identified in the PCA (Figure 5A,B), where rainwater primarily reflects variability in atmospheric composition, whereas stream-water samples follow gradients of mineralisation and streamflow. Together, these findings demonstrate that seasonal hydrological regulation is a fundamental component of the conceptual model proposed in this study (Figure 6), linking atmospheric deposition with the progressive hydrochemical evolution of stream water in Mediterranean forested karst catchments.

### 3.3. Environmental Implications and Future Perspectives

The present study provides new insight into hydrochemical functioning in Mediterranean forested karst catchments by integrating atmospheric deposition and stream-water hydrochemistry within a single process-based framework. Unlike most previous studies, which have generally focused either on atmospheric deposition in Mediterranean forests [4,9,10,17,25] or on hydrochemical evolution in Mediterranean karst waters [30,31,32,33,34,35,54], this study simultaneously investigated both water types within the same Mediterranean karst catchment over a three-year monitoring period. This integrated approach enabled identification of the dominant processes governing the transformation of atmospheric inputs during their passage through the catchment and provides a more comprehensive framework for interpreting hydrochemical functioning in Mediterranean carbonate environments.

The conceptual model proposed in this study synthesises the principal findings of this work. By integrating evidence derived from major-ion chemistry, ionic ratios, sea-salt/non-sea-salt fractionation, seasonal analyses, correlation analysis and principal component analysis, the model demonstrates how atmospheric inputs are progressively modified before being expressed in stream-water chemistry. Because the interaction between atmospheric deposition, carbonate lithology and Mediterranean climatic conditions characterises many forested karst landscapes throughout the Mediterranean Basin, the proposed framework may also be applicable to other Mediterranean carbonate catchments with comparable geological and climatic settings [22,30,31,32,33,54] and is consistent with the broader hydrogeochemical concepts describing the evolution of natural waters [18,36,37].

The findings are also relevant for predicting the response of Mediterranean karst catchments to future environmental change. Climate projections consistently indicate increasing air temperatures, more frequent and prolonged droughts, reduced summer precipitation and greater variability in rainfall regimes across the Mediterranean region [60,61,62]. The seasonal patterns identified in this study (Figure 3, Figure 4 and Figure 5A,B) suggest that these changes are likely to intensify the hydrochemical processes observed in the Žrnovnica catchment. Prolonged dry periods may promote the accumulation of marine aerosols, mineral dust and atmospherically deposited solutes on vegetation canopies and soil surfaces, followed by their episodic mobilisation during subsequent rainfall events, consistent with observations from Mediterranean environments [9,63]. At the same time, reduced groundwater recharge and longer groundwater residence times are expected to enhance carbonate dissolution and stream-water mineralisation, whereas more intense rainfall events may strengthen hydrological connectivity and increase the transport of atmospherically derived solutes through the catchment [64,65,66]. Consequently, future hydrochemical responses of Mediterranean karst catchments are likely to depend not only on changes in atmospheric deposition but also on the interaction between climatic variability and internal catchment processes. Understanding these interactions is therefore essential for predicting future changes in the quality and sustainability of Mediterranean karst water resources.

Although the present study provides a comprehensive assessment of the relationships between rainwater and stream-water chemistry, several limitations should be acknowledged. This investigation was conducted within a single Mediterranean forested karst catchment dominated by Aleppo pine, and therefore the magnitude of the observed hydrochemical responses may differ among catchments with contrasting lithology, vegetation structure, land use or climatic conditions. Furthermore, this study focused on major ions and basic physicochemical parameters. Future investigations integrating high-frequency sampling, groundwater monitoring, stable isotope tracers, dissolved organic carbon, trace elements, groundwater age indicators and atmospheric back-trajectory analyses would provide additional insight into groundwater flow paths, potential time lags between precipitation and stream-water responses, the sources and transport of dissolved constituents, and the relative contributions of marine aerosols, Saharan dust and regional anthropogenic emissions to catchment hydrochemistry, while also enabling the process-based interpretation proposed in this study to be evaluated more directly.

Future research should therefore extend this integrated monitoring approach to multiple Mediterranean karst catchments while combining long-term hydrochemical observations with isotope techniques, hydrological modelling and process-based biogeochemical analyses. Such comparative investigations would improve understanding of how differences in geology, vegetation and climate regulate hydrochemical functioning across the Mediterranean Basin and would strengthen predictions of catchment responses to future changes in atmospheric deposition and climate.

## 4. Materials and Methods

### 4.1. Study Sites

**Figure 7:**
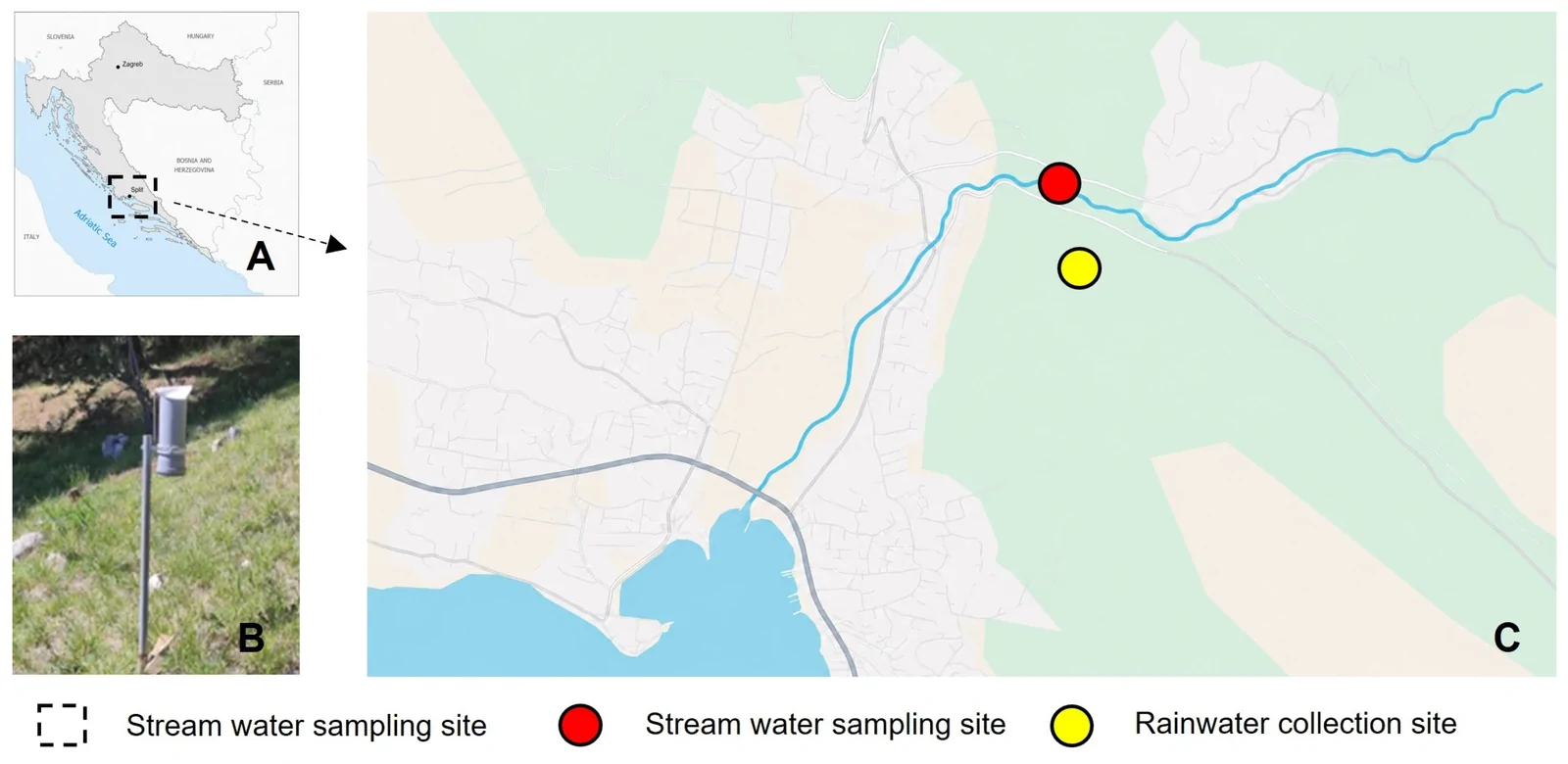
Study area and sampling locations: (**A**) location of the Žrnovnica catchment; (**B**) rain-water collector used for precipitation sampling; (**C**) map of the Žrnovnica stream, stream-water sampling site and rainwater collection site.

This study was conducted in the Žrnovnica catchment located within the Eastern Adriatic and the coastal Dinarides (Figure 7). The terrain is characterized by a typical NW–SE Dinaric structural orientation and well-developed karst geomorphology. The geological substrate is dominated by highly karstified Mesozoic and Eocene carbonate rocks characterized by high permeability and limited soil cover. These conditions result in the near absence of perennial surface watercourses [35,67,68]. Common karst landforms in the catchment include dolines, dry valleys, sinkholes, pits, and caves. The predominant soil type is brown soil on limestone, classified as Calcocambisols [68]. The Žrnovnica stream represents the main local water source for nearby settlements and agricultural areas [38]. Phytogeographically, the catchment belongs to the Mediterranean vegetation region, specifically the montane–littoral belt [68]. The dominant forest species in the catchment is Aleppo pine (*Pinus halepensis*) which is well adapted to hot, dry summer conditions. The climate is Mediterranean characterized by mild, wet winters and hot, dry summers, with most rainfall occurring in short, intense autumn and winter events [69,70]. The mean annual air temperature in the study area is 16.1 °C, while mean annual rainfall is 782.8 mm.

### 4.2. Rainwater and Stream-Water Sampling and Analysis

Sampling, measurements, and analyses of rainwater were performed according to the International Cooperative Programme on Assessment and Monitoring of Air Pollution Effects on Forests (ICP Forests) manuals [71]. Rainwater was collected using three collectors installed in the open field. Each collector consisted of a high-density polyethylene funnel with a surface area of 200 cm^2^ connected to a collection bottle. An inert polyethylene sieve (1 mm mesh size) was loosely placed on the neck of the funnel to prevent contamination by insects and debris. The collectors were installed at a height of 1.5 m above ground. To minimize light exposure and evaporation, the collection bottles were placed inside PVC tubes. The upper part of the collector was designed with smooth and continuous surfaces, without joints or ridges, to reduce water retention and evaporation losses, and to ensure rapid drainage of rainwater into the collection bottle [71]. After each sampling event, the collectors were inspected to remove debris and to ensure that the equipment remained level and unobstructed. Stream water was sampled from the Žrnovnica stream in the immediate vicinity of the rainwater collection site. Sampling followed established guidelines to avoid disturbance of the streambed and to prevent inclusion of surface films [72]. Continuous streamflow data were obtained from the Croatian national water authority “Hrvatske vode”. Rainwater and stream water were sampled simultaneously at approximately 15-day intervals from March 2020 to December 2022. A total of 33 paired rainwater and stream-water samples were collected and included in the statistical analyses. All samples were collected using 200 mL bottles, stored at 4 °C, and transported to the laboratory for chemical analysis [71]. Electrical conductivity (EC) and pH were measured in unfiltered samples using a Mettler Toledo MC 226 EC meter and a Mettler Toledo MP 230 pH meter, respectively (Mettler Toledo, Columbus, OH, USA).Ion concentrations and total alkalinity were determined in samples filtered through 0.45 µm membrane filters. Concentrations of calcium (Ca^2+^), magnesium (Mg^2+^), sodium (Na^+^), potassium (K^+^), ammonium (NH_4_^+^), sulphate (SO_4_^2−^), nitrate (NO_3_^−^), and chloride (Cl^−^) were quantified using ion chromatography (Dionex DX500, Dionex Corporation, Sunnyvale, CA, USA) with PeakNet software, version 5,following the analytical procedures described by ICP Forests [71].

### 4.3. Calculations

Ion ratios were computed from molar concentrations of major ions to identify dominant geochemical processes. Ion ratio calculations followed established approaches in atmospheric chemistry and marine aerosol [12,47,73]. Sea-salt (ss) and non-sea-salt (nss) fractions of ions were determined following [12], using sodium (Na^+^) as a conservative tracer of marine aerosol input and applying seawater reference ratios [12].

The sea-salt component of each ion was calculated as follows:

Xss = (X/Na^+^) seawater × Na^+^ measured (1)

The non-sea-salt fraction was obtained as follows:

Xnss = Xtotal − Xss(2)

where X represents the concentration of the ion of interest.

Relative contributions of sea-salt and non-sea-salt components were expressed as percentages of the total ion concentration.

### 4.4. Data Analysis

Descriptive statistics were calculated separately for rainwater and stream water for all measured parameters. Normality was assessed using the Shapiro–Wilk test. Because most variables deviated from normal distributions, non-parametric statistical tests were used for comparisons between groups. Differences in ion concentrations and physicochemical parameters between rainwater and stream water were analysed using the Mann–Whitney U test, with statistical significance set at *p* < 0.05. For seasonal analysis, rainwater and stream-water data were grouped according to meteorological seasons (winter: December, January and February; spring: March, April, and May; summer: June, July and August; autumn: September, October and November). Seasonal differences were evaluated using the Kruskal–Wallis test, and when significant differences were detected (*p* < 0.05), Statistica’s post hoc multiple comparison procedures were applied. Spearman’s rank correlation coefficients (ρ) were calculated to assess relationships between corresponding parameters in rainwater and stream water. Correlations were evaluated for the entire study period (2020–2022) and separately for each season, with statistical significance assessed at *p* < 0.05. Principal Component Analysis (PCA) was performed using the correlation matrix of standardized variables to identify multivariate patterns, dominant hydrochemical gradients and potential source signatures in rainwater and stream-water chemistry. PCA was used as an exploratory multivariate technique and biplots were used to visualize variable loadings and interrelationships. Given the relatively limited sample size, PCA results were interpreted cautiously and in conjunction with descriptive statistics, seasonal analyses and Spearman’s correlation analysis rather than as a stand-alone confirmatory analysis. All statistical analyses were performed using Statistica 14.0 (TIBCO Software Inc., Palo Alto, CA, USA) [74].

## 5. Conclusions

This study provides new evidence that rainwater and stream water represent successive stages of hydrochemical evolution within an Aleppo-pine-dominated Mediterranean karst catchment, with atmospheric inputs being progressively modified during their passage through the catchment. While rainwater primarily reflected mixed atmospheric sources, stream-water chemistry was predominantly controlled by carbonate weathering, hydrological flow paths, and biogeochemical processes operating within the catchment. This transformation was reflected in substantially higher stream-water mineralisation, including an approximately tenfold enrichment of Ca^2+^ and Mg^2+^ relative to rainwater. Seasonal hydrological conditions emerged as an important factor regulating these transformations, with summer low-flow conditions associated with enhanced stream-water mineralisation and wetter winter conditions promoting dilution and groundwater renewal. However, the limited synchronous correspondence between rainwater and stream-water chemistry should not be interpreted as evidence of an absence of hydrological connectivity, because potential time-lagged responses associated with groundwater storage and release cannot be resolved by the present sampling design.

While many previous Mediterranean studies have investigated atmospheric deposition or stream-water hydrochemistry separately, this study integrated both components within the same Mediterranean forested karst catchment, allowing their relationship to be evaluated within a single catchment-scale framework.

The findings improve our understanding of hydrochemical functioning in the Žrnovnica catchment and may be particularly relevant to similar Mediterranean karst forested catchments along the eastern Adriatic coast, where comparable climatic, geological and vegetation conditions prevail. This findings provide a basis for assessing potential hydrochemical responses to changing atmospheric and climatic conditions and may contribute to the sustainable management of karst water resources in this region.

## Figures and Tables

**Table 1:** Descriptive statistics and comparison of major ion concentrations. pH, electrical conductivity (EC) and total alkalinity in rainwater and stream water during 2020–2022.

Parameter	Rainwater					Stream Water					Ratio (SW/RF)	*p*-Value
	Mean (±SD)	Min	Max	Median	IQR	Mean (±SD)	Min	Max	Median	IQR		
pH	6.6 (±0.7)	5.2	7.9	6.5	0.7	8.2 (±0.3)	7.5	9.0	8.2	0.3	1.3	**<0.001**
EC	23.76 (±16.74)	2.15	91.20	19.63	15.56	337.83 (±107.18)	136.60	747.00	327.00	74	16.66	**<0.001**
Ca^2+^	9.23 (±7.41)	1.71	32.89	6.22	5.80	65.68 (±28.22)	9.76	120.12	65.08	51.448	10.47	**<0.001**
Mg^2+^	0.74 (±0.71)	0.16	2.75	0.43	0.52	5.12 (±2.69)	0.52	13.09	4.80	3.055	11.26	**<0.001**
Na^+^	2.04 (±1.40)	0.50	5.55	1.38	2.07	10.19 (±6.23)	2.92	24.99	7.79	10.2352	5.64	**<0.001**
K^+^	0.53 (±0.42)	0.13	1.91	0.41	0.37	1.03 (±0.79)	0.10	4.16	0.86	0.5185	2.09	**0.004**
NH_4_^+^	0.28 (±0.37)	0.002	1.84	0.16	0.31	0.24 (±0.28)	0.00	0.99	0.10	0.438	0.63	0.323
SO_4_^2−^	1.93 (±1.24)	0.19	6.15	1.83	0.79	7.56 (±3.79)	1.85	15.51	7.44	5.366	4.06	**<0.001**
NO_3_^−^	0.76 (±0.64)	0.01	2.39	0.54	0.68	0.95 (±0.86)	0.00	3.55	0.78	1.0571	1.44	0.509
Cl^−^	3.46 (±2.18)	1.00	10.51	2.74	2.00	15.75 (±12.18)	3.35	48.23	10.52	8.39	3.84	**<0.001**
T. Alk	0.39 (±0.20)	0.16	0.80	0.34	0.24	3.36 (±0.69)	1.28	4.40	3.40	0.81	10.00	**<0.001**

Values are presented as mean ± standard deviation (SD), minimum (Min), maximum (Max), median, and interquartile range (IQR). The ratio (SW/RF) represents the ratio of median stream water (SW) to rainwater (RF) values for pH, EC and ion concentrations. Statistically significant differences (Mann–Whitney U test, *p* < 0.05) are indicated in bold. EC—electrical conductivity (µS cm^−1^); T. Alk—total alkalinity (mg L^−1^); ion concentrations are expressed in mg L^−1^.

**Table 2:** Spearman’s rank correlation coefficients (ρ) between the same parameters in rainwater and stream water for the entire study period (2020–2022) and by season (spring, summer, autumn and winter). Statistically significant correlations (*p* < 0.05) are shown in bold.

Parameter (Rainwater—Stream Water)	2020–2022		Spring		Summer		Autumn		Winter	
	ρ	*p*	ρ	*p*	ρ	*p*	ρ	*p*	ρ	*p*
pH	0.242	0.175	−0.071	0.867	−0.600	0.400	0.191	0.573	0.085	0.815
EC	0.081	0.654	−0.071	0.867	−0.400	0.600	0.436	0.180	0.164	0.651
Ca^2+^	0.257	0.148)	**−0.738**	**0.037**	0.600	0.400	**0.681**	**0.021**	0.042	0.907
Mg^2+^	0.093	0.607	0.476	0.233	1.000	-	0.064	0.853	0.515	0.128
Na^+^	−0.202	0.259	0.357	0.385	0.800	0.200	−0.064	−0.853	−0.018	0.960
K^+^	−0.012	0.948	0.262	0.531	−0.800	0.200	0.027	0.937	0.042	0.907
NH_4_^+^	0.122	0.500	0.342	0.408	−0.200	0.800	−0.109	0.750	0.614	0.059
SO_4_^2−^	0.118	0.514	0.071	0.867	0.800	0.200	−0.009	0.979	0.176	0.627
NO_3_^−^	**0.370**	**0.034**	−0.381	0.352	−0.400	0.600	0.391	0.235	0.442	0.200
Cl^−^	−0.142	0.431	0.571	0.139	−0.200	0.800	−0.364	0.272	−0.224	0.533

Because only four paired observations were available during summer, the corresponding Spearman’s correlation coefficients should be interpreted with caution.

## Data Availability

Data are contained within the article.
